# Precision and Disclosure in Text and Voice Interviews on Smartphones

**DOI:** 10.1371/journal.pone.0128337

**Published:** 2015-06-10

**Authors:** Michael F. Schober, Frederick G. Conrad, Christopher Antoun, Patrick Ehlen, Stefanie Fail, Andrew L. Hupp, Michael Johnston, Lucas Vickers, H. Yanna Yan, Chan Zhang

**Affiliations:** 1 Department of Psychology, New School for Social Research, The New School, New York, New York, United States of America; 2 Institute for Social Research, University of Michigan, Ann Arbor, Michigan, United States of America; 3 Joint Program in Survey Methodology, University of Maryland, College Park, Maryland, United States of America; 4 Loop AI Labs, San Francisco, California, United States of America; 5 Interactions, New York, New York, United States of America; 6 Design & Technology, Parsons School of Design, The New School, New York, New York, United States of America; 7 Media and Public Opinion Research Center, Fudan University, Shanghai, China; Texas A&M University, UNITED STATES

## Abstract

As people increasingly communicate via asynchronous non-spoken modes on mobile devices, particularly text messaging (e.g., SMS), longstanding assumptions and practices of social measurement via telephone survey interviewing are being challenged. In the study reported here, 634 people who had agreed to participate in an interview on their iPhone were randomly assigned to answer 32 questions from US social surveys via text messaging or speech, administered either by a human interviewer or by an automated interviewing system. 10 interviewers from the University of Michigan Survey Research Center administered voice and text interviews; automated systems launched parallel text and voice interviews at the same time as the human interviews were launched. The key question was how the interview mode affected the *quality* of the response data, in particular the precision of numerical answers (how many were not rounded), variation in answers to multiple questions with the same response scale (differentiation), and disclosure of socially undesirable information. Texting led to higher quality data—fewer rounded numerical answers, more differentiated answers to a battery of questions, and more disclosure of sensitive information—than voice interviews, both with human and automated interviewers. Text respondents also reported a strong preference for future interviews by text. The findings suggest that people interviewed on mobile devices at a time and place that is convenient for them, even when they are multitasking, can give more trustworthy and accurate answers than those in more traditional spoken interviews. The findings also suggest that answers from text interviews, when aggregated across a sample, can tell a different story about a population than answers from voice interviews, potentially altering the policy implications from a survey.

## Introduction

The growing use of smartphones is transforming how people communicate. It is now ordinary for people to interact while they are mobile and multitasking, using whatever mode—voice, text messaging, email, video calling, social media—best suits their current purposes. People can no longer be assumed to be at home or in a single place when they are talking on the phone, if they are willing to talk on the phone at all as opposed to texting or using another asynchronous mode of communication [1]. And they may well be doing other things while communicating more than they would have been even a few years ago.

This transformation is challenging the basis of how we gather essential information about society: how we measure our health, employment, consumer confidence, crime, education, and many other human activities. Modern social measurement depends on face-to-face (FTF) and landline telephone surveys, and more recently on self-administered web surveys on personal computers. As FTF and landline telephone communications change, it is possible that current methods will not be sustainable [2]. But the critical need for accurate data about the population persists; effective public policy and private sector strategy depend on credible measurement of people’s opinions and behaviors. For example, world economies and US electoral politics can be significantly affected by the US unemployment rate reported each month from the Current Population Survey, a government-sponsored survey with a sample of 60,000 households per month. As another example, policies on disease prevention, health insurance, and risk-related behaviors depend on surveys such as the Behavioral Risk Factor Surveillance System (BRFSS), in which a consortium of US states and the Centers for Disease Control and Prevention interview more than 400,000 US households per year to track health and disease trends. Any challenges to the accuracy of such data threaten our ability to understand ourselves collectively and create effective policy.

In the study reported here, we explored how members of the public report information about themselves in a survey when they are randomly assigned to respond in one of the new communication modes they now use every day, but which have not yet been used in social science and government surveys on a large scale. Our experimental design contrasts two factors that reflect the diversity in communication modes available on a single mobile device (in our case the iPhone): the *medium* of communication, voice vs. text messaging, and the *interviewing agent*, a human interviewer vs. an automated interviewing system. This leads to four modes of interviewing: Human Voice (telephone), Human Text (text message interview administered by an interviewer), Automated Voice (telephone interview administered by an automated system), and Automated Text (text message interview administered by an automated system). (The Automated Voice system is a version of what is known as Interactive Voice Response [IVR] in polls, market research, and other application areas, most often with touchtone response; see [3] on speech IVR systems). Each respondent was randomly assigned to one of these modes and answered on their own iPhone.

Our primary question is how these factors and the modes of interviewing they comprise affected the quality of survey data, as well as respondents’ subjective experience. We also examine what else respondents did while answering questions in these modes—whether they were multitasking and/or mobile—and how this affected the quality of their answers in the different modes. Because we measure survey responding on the same device for all respondents (as opposed to including other platforms such as Android or Windows), we can make fair experimental comparisons, even if the same modes could be deployed on other devices. Because respondents all used the uniform iPhone interface, any differences in responding across the modes cannot be because of platform differences. Because respondents used native apps on the iPhone—the Phone app or the Messages app—which they knew well and used for daily communication (as opposed to answering a web survey in a browser on the iPhone, or a specially designed survey app that they would need to download), any differences in responding across the modes are unlikely to have resulted from differential experience with the modes.

We examine data quality in these four modes by measuring the extent to which respondents’ answers were careful and conscientious (i.e., the extent to which respondents were not taking mental shortcuts or “satisficing”, see [4]–[5]), and the extent to which respondents were willing to disclose sensitive information. We measure thoughtfulness in answering questions that require numerical responses by looking at the percentage of answers that were precise (that is, not "heaped" or rounded by ending in a zero or a five); unrounded answers are more likely to result from deliberate, memory-based thought processes than estimation (see [6]–[7]), and they are more likely to be accurate in answers to objective factual questions [8]. We measure care in answering multiple questions that use the same response scale—from “strongly favor” to “strongly oppose”—by looking at the percentage of responses that were different from each other; the general view is that some variation across the responses (as opposed to “straightlining,” where the same response is given again and again) is likely to reflect more conscientious or thoughtful responding [9]. We use increased disclosure of sensitive information (e.g., more reported lifetime sexual partners, more reported alcohol use) as evidence of improved data quality, consistent with the evidence in survey research that more embarrassing answers are more likely to be true [10]–[12].

### How might texting affect survey data quality?

Little is yet known about how survey responding differs between mobile voice and text messaging interviews. Will people respond less thoughtfully in text because it is often used for casual communication, or more thoughtfully because there is less time pressure to respond? Will they respond less honestly because they aren’t hearing or speaking directly to a human interviewer, or more honestly because they feel less inhibited without spoken contact? Will the lasting visual record of text messages, which others might see, make people answer more honestly because they feel accountable, or less honestly because they feel embarrassed?

Texting and speaking differ in fundamental ways that could affect both precision and disclosure in surveys, as Table 1 outlines. In addition to leaving a persistent visual record of what has been communicated, texting is less synchronous than speaking; the delay between successive text messages can be hours or even days. Even when the back-and-forth of texting is quick, it still does not allow simultaneous production and reception in the way that speaking does, nor does it allow precisely timed requests for clarification or feedback during the partner’s speech, because utterances arrive fully formed. In general, the rhythm of texting is quite different than the rhythm of speech: speakers in conversation are typically expected to respond immediately or to account for any delays (e.g., [13]–[14]), while the same expectations do not necessarily hold in text. In text, delay or complete lack of response doesn’t necessarily signal a problem in the way it does in speech.

**Table 1:** Voice vs. text on smartphones.

Property	Voice	Text
Synchrony	Fully synchronous	Less or asynchronous
Medium	Auditory	Visual
Language	Spoken/heard	Written/read
Conversational structure	Turn-by-turn, with potential for simultaneous speech	Turn-by-turn, rarely but possibly out-of-sequence
Persistence of turn	No	Yes
Persistence of entire conversation	No	Yes, threaded
Social presence of partner	Continuous (auditory) presence	Intermittent evidence from content of texts; no additional evidence between texts
Nonverbal cues of emotional state and intentions	Always present: speech always has pitch and timing	Only present if added by sender through words (e.g., "LOL"), orthography (e.g., capital letters, punctuation) or emoticons (e.g., ☺)
Character of multitasking	Often simultaneous, especially when hands free, unless other task involves talking	Switching often required between texting and other tasks
Impact of environmental conditions	Potential (auditory) interference from ambient noise	Potential (visual) interference from visual glare
Impact of nearby others	Others may hear speaker’s side of conversation; potential audio interference from others’ talk	Others unlikely to see conversation on screen, though possible
Required connection to network (cellular or wifi)	Must be continuous	Can be intermittent

The effort required to communicate—to produce, comprehend and repair utterances—in voice and text also differs. In general, talking and typing require different mental and social processes [15], and the style of communication that has evolved in texting can be abbreviated (stemming in part from earlier character limits on the length of text messages) and less formal than other written modes [16]. In most cases people find it easy to talk and harder to type, although this may vary for different people, cohorts, and different mobile environments; for example, it may be easier to text than talk when it is noisy, and particularly hard to text when there is visual glare. Because of the lag between text messages, people texting may be more likely to shift their attention to other tasks, which means that to continue a text thread they must return their gaze and attention to the smartphone screen. In other words, the effort for multitasking (whether that means alternating between tasks or performing them simultaneously) can differ significantly between text and voice—texting while walking is harder (and less safe) than talking while walking. Additional effort in dialogue management can be required in texting if messages are received out of sequence because of network performance problems.

More difficult to quantify is how different the social presence of the communicative partner is in texting. Texting *feels* different; there is no continuous (auditory) evidence about the presence of one’s partner—less of a sense that the partner is there (see, e.g., [17]–[18])—and less chance of interruption or feedback during an utterance. Text also doesn’t have as rich a palette of intonation cues, which can give a sense of a communication partner’s mental and emotional states, evaluative judgment, or attentiveness (though see [16] for discussion of the "paralinguistic restitution" that texters use to communicate nonverbal content).

These differences could easily affect both precision and disclosure in surveys. For precision, texting reduces demand to respond immediately, and may enable respondents to take more time thinking about their answers and to respond more precisely. Alternatively, respondents might engage in more task-switching while texting, leading them to answer less precisely because the mental resources required for task switching diminish their processing ability. And the reduced social presence of the interviewer in text could lead respondents to feel less accountable for their answers, which could lead to less precision.

For disclosure, texting offers a combination of features that could lead respondents to report more embarrassing information than when speaking: the asynchrony and reduced social presence of the interviewer give less immediate evidence of an interviewer’s reaction to answers, and possibly more time to grow comfortable with the potential consequences of disclosure. Also, texting an answer to a sensitive question might feel more “private” than speaking it out loud. On the other hand, respondents might disclose less in text if they worry about the possibility of others eventually discovering the answers on their phone, on synced computers, or in service providers’ databases. The asynchrony of texting could also give respondents more time to devise answers that are evasive or less truthful.

### How might automated interviewing systems affect survey data quality on smartphones?

As for automated interviewing on smartphones, it is well known that people respond differently to “self-administered” surveys delivered by automated systems than by human interviewers, and this affects respondents’ care in answering (for better [19] or worse [20]) and increases disclosure (e.g., [10], [21]). Our question is whether these effects are also observed in surveys administered on a smartphone, where respondents can be mobile, in differently distracting environments, and potentially less private than when answering on desktop computers or landline telephones. It is also unclear whether these effects will extend to automated surveys administered with the turn-taking structure of voice and text, as opposed to web surveys. If, for example, respondents feel less social presence (and thus accountability) with a text interviewer than a voice interviewer, then perhaps automation won’t matter in text.

Regarding conscientious responding, the evidence in traditional survey modes is mixed. People seem to skip fewer questions in web surveys than in telephone interviews [20], but they sometimes straightline more [20] and sometimes less [19], [22]–[23]. It is unclear whether this happens because the web surveys are automated (they lack an interviewer) or the fact that the interaction is textual, or both. Our texting modes allow us to examine this directly.

As for disclosure, people are known to report more sensitive information—more sex partners, more substance use, poorer academic performance—in automated surveys than to human interviewers (e.g., [10], [21]). The usual explanation for this is that the privacy and anonymity of automated interviews reduce concern about being judged and one’s answers revealed [11], [24]–[25], though what may be particularly important is the lack of the interviewer’s face [26]. If these effects replicate in text and voice on smartphones, then we should see greater disclosure in Automated Voice and Text interviews than Human Voice and Text interviews. Whether the effects will be the same in voice and text is less clear. If social presence is lower in text than voice (even with a human interviewer), then automation may not affect disclosure in text at all. And given that with a mobile device people can choose when and where they respond, it is unknown whether the privacy concerns that promote disclosure with automated systems are still relevant.

## Materials and Methods

A convenience sample of iPhone users was recruited from a variety of online sources to participate in a survey on their iPhone for a $20 iTunes gift code incentive. They were then randomly assigned to one of the four interview modes (Human Voice, Human Text, Automated Voice, Automated Text). All procedures were approved by the Health Sciences and Behavioral Sciences Institutional Review Boards at the University of Michigan and the Institutional Review Board at The New School.

### Experimental Design

**Fig 1:**
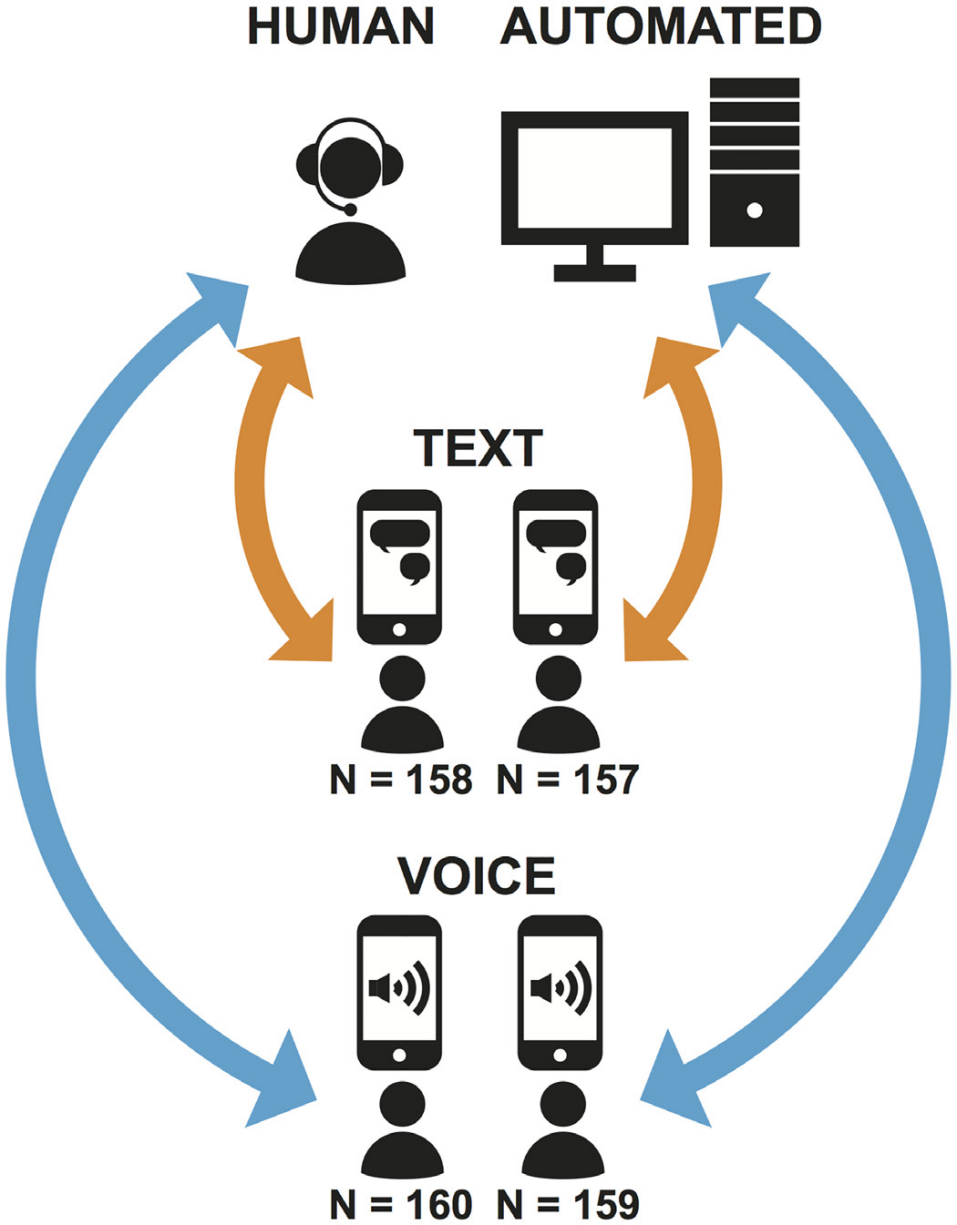
Experimental design and procedure.

The experimental design contrasted two factors, interviewing medium (voice vs. text) and interviewing agent (human vs. automated), creating four modes in a 2x2 design (see Fig 1). The four modes were implemented to be as comparable to each other as possible so as to allow clean comparisons between voice and text as well as between human and automated interviewing agents. In all modes questions were presented following standardized protocols, with precisely the same question wording, the same turn-taking (conversational back-and-forth) structure between (automated or human) interviewer and respondent, and the same protocols for handling any speech or text by respondents other than acceptable answers (handling requests for clarification, responses that did not match the response options, responses that could not be perceived or understood, and out-of-scope commentary). In voice interviews respondents answered by talking; in text interviews respondents were instructed to answer by texting a single character corresponding to a response option (Y/N, a letter [e.g., a, b, c], or a number).

The human interviewers used a custom-designed desktop interface connected to a custom-designed case management system, which randomly assigned participants to interview modes and interviewers and stored the survey data. The interface for voice presented interviewers with the questions for them to read aloud, and they selected or typed the answers they heard. In the text interface interviewers selected, edited or typed questions and prompts and clicked to send the text messages, which were routed through a third party provider (Aerialink); they then entered respondents’ texted answers through the interface. 10 professional interviewers at the University of Michigan Survey Research Center carried out both voice and text interviews; they all administered interviews in both modes.

For the automated voice interviews, a custom speech dialogue system was created using AT&T’s Watson speech recognizer and the Asterisk telephony gateway; questions and prompts were presented by a recorded female human interviewer, and respondents’ speech (not touchtone) was recognized by Watson; answers were automatically stored (see [27] for details). For the automated text interviews, a custom text dialogue system was created; text messages were also routed through Aerialink, and respondents’ texted answers were automatically stored in the database (see S1 Fig for a system diagram). The introductory material for the Automated Text interviews explicitly labeled the interview as automated (“This is an automated interview from the University of Michigan”), while the comparable materials in the Human Text interviews identified the interviewer (“My name is < first name, last name> from the University of Michigan”). All messages sent in the Automated Text interviews were pre-scripted, while the human text interviewers could individualize the scripted materials (other than the survey questions) if they chose; they could also generate original message content if they deemed it necessary.

### Materials and Procedures

**Fig 2:**
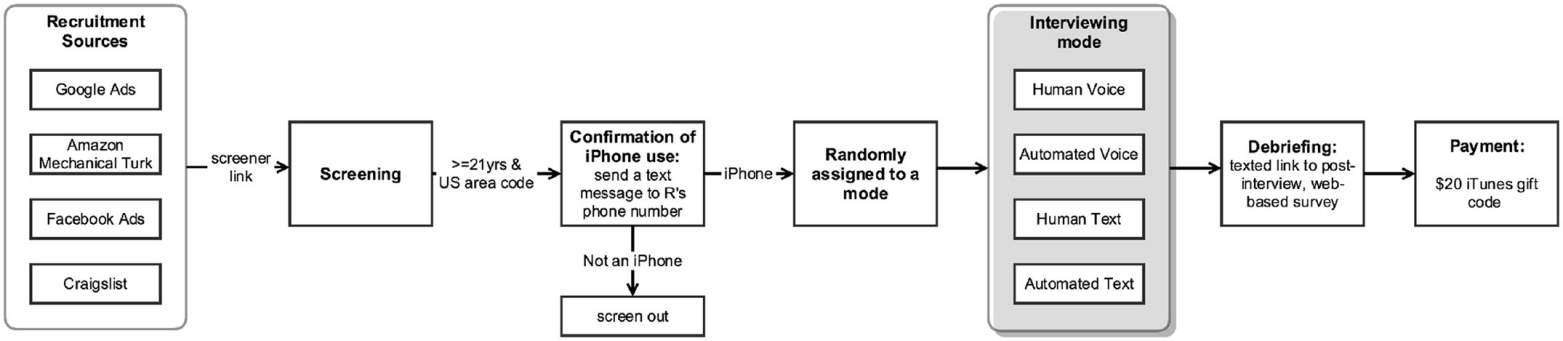
Procedures.

Fig 2 summarizes the sequence of steps that participants experienced.

#### Recruitment and Screening

iPhone users were recruited from Google AdWords (31.0%), Amazon Mechanical Turk (60.9%), Facebook Ads (0.3%) and Craigslist (7.8%) (see [28] for analyses of the impact of these recruitment sources on sample composition). They followed a link from the recruitment source to a browser-based screener questionnaire that asked them for their zip code, date of birth, education, income, gender, ethnicity, race, telephone number, service provider, voice minutes and text message plan, whether they were the sole user of this phone, and time zone. The screener asked them to explicitly consent to participate in a research study by clicking a check box. If the participants reported being 21 years of age and over, and if their phone number did indeed have a US area code, they were sent a text message to which they replied so that we could verify that their device was an iPhone. If their device was verified as an iPhone, they were randomly assigned to one of the four modes of interviewing.

#### Interview

All interviews were started with either a safe-to-talk or safe-to-text question and could only continue if respondents indicated that it was in fact safe. The same 32 questions from major US social surveys and methodological studies were asked in each mode (see S1 Table, which lists the questions in the order they appeared in the interview, along with their source). Fifteen questions were selected to allow measurement of conscientious responding: 8 questions requiring numerical responses which could later be classified as precise or rounded (divisible by 5) numbers (e.g., number of movies seen last month, number of apps on iPhone) and a battery of 7 questions that all used the same 5-point response scale (from “strongly favor” to “strongly oppose”) allowing measurement of straightlining (generally defined as selecting the same response options for all or most questions in a battery). 15 questions with more or less socially desirable answers were selected to allow measurement of disclosure, in that they had more and less socially desirable answers (e.g., number of sex partners, lifetime marijuana use, frequency of reading the newspaper, etc.). A number of these questions were known to have produced differences in conscientious responding or disclosure in conventional survey modes.

#### Debriefing

After completing the interview, respondents were sent a text message with a link to the post-interview online debriefing questionnaire. This questionnaire asked about the presence of others during the interview, interference from environmental conditions (background noise, glare), multitasking, intrusiveness of the questions, preference for mode of future interviews among the four in this study, ease of completing the interview, and satisfaction with the interview. Upon completion of the post-interview debriefing questionnaire, a text message was sent with a $20 iTunes gift code as payment for participation.

### Participants

The data reported here are based on the responses of the 634 respondents who completed the interview and the on-line debriefing questionnaire between March and May 2012 (see Table 2 for completion rates).

**Table 2:** Participation, response rates and break-off rates.

Mode	Invitations	Started Interview	Completed Interview	ResponseRate^a^	Break-off Rate^b^	Completed Online Debriefing
**Human Voice**	316	169	164	**51.90%**	2.96%	160
**Automated Voice**	414	187	162	**39.13%**	13.37%	159
**Human Text**	227	176	163	**71.81%**	7.39%	158
**Automated Text**	325	185	159	**48.92%**	14.05%	157
Total	1282	717	648	**50.55%**	9.62%	634

^a^The response rate (known as AAPOR RR1 [29]) is calculated as the number of complete interviews divided by the number of invitations.

^b^The break-off rate is calculated as the number of people who dropped off during the survey divided by the number of people who started.

### Respondent demographics

The respondents were somewhat younger and less affluent than US national iPhone users at the time [30] but across the four modes they did not differ reliably in age, education, income, gender, ethnicity, race, or cell phone carrier (see Table 3).

**Table 3:** Respondent demographics across four modes.

Demographic variables		Voice		Text				
		Human	Automated	Human	Automated	Chi-square statistics	Degrees of freedom	*p*
Age^a^						7.833	12	0.798
	*21–34 years*	56.30%	62.70%	57.00%	64.70%			
	*35–44 years*	15.00%	13.90%	17.10%	10.30%			
	*45–54 years*	12.50%	12.00%	12.70%	11.50%			
	*55–64 years*	13.10%	10.10%	9.50%	9.60%			
	*65 years and older*	3.10%	1.30%	3.80%	3.90%			
Education						7.275	12	0.839
	*High school or less*	7.50%	5.70%	8.90%	7.60%			
	*Some college*	28.80%	28.30%	24.10%	28.70%			
	*Associate/Vocational/Technical*	11.90%	8.10%	9.50%	13.40%			
	*Bachelor's degree*	28.10%	37.10%	33.50%	29.90%			
	*Master/Professional/PhD*	23.80%	20.10%	24.10%	20.40%			
Income						10.861	12	0.541
	*$25000 or less*	17.50%	18.20%	13.90%	16.60%			
	*$25000 to $50000*	21.90%	25.80%	24.70%	29.90%			
	*$50000 to $75000*	16.30%	17.60%	22.80%	18.50%			
	*$75000 or more*	31.90%	32.10%	30.40%	29.30%			
	*Decline to answer*	12.50%	6.30%	8.20%	5.70%			
Gender						2.533	3	0.469
	*Female*	41.90%	49.70%	49.40%	47.80%			
	*Male*	58.10%	50.30%	50.60%	52.20%			
Hispanic origin						3.896	3	0.273
	*No*	88.10%	93.70%	90.50%	93.00%			
	*Yes*	11.90%	6.30%	9.50%	7.00%			
Race						5.367	9	0.801
	*White*	76.90%	81.80%	76.60%	79.00%			
	*African American*	9.40%	3.80%	7.00%	5.70%			
	*Asian*	5.60%	6.90%	7.00%	5.70%			
	*Other*	8.10%	7.60%	9.50%	9.60%			
Carrier						3.731	6	0.713
	*AT&T*	60.00%	62.30%	64.60%	66.90%			
	*Sprint*	10.00%	7.60%	10.10%	10.20%			
	*Verizon*	30.00%	30.20%	25.30%	22.90%			

^a^ Age values are missing for two respondents (n = 632 instead of 634).

## Results

We report findings based on the responses recorded by the human interviewers (in Human Voice and Human Text) and by the automated systems (in Automated Voice and Automated Text). In the Automated Voice mode, the system correctly recognized the answers in 95.6% of the responses; the patterns of results do not differ if we use human annotations, the presumed gold standard (see [27]).

### Data quality: Conscientious responding

**Fig 3:**
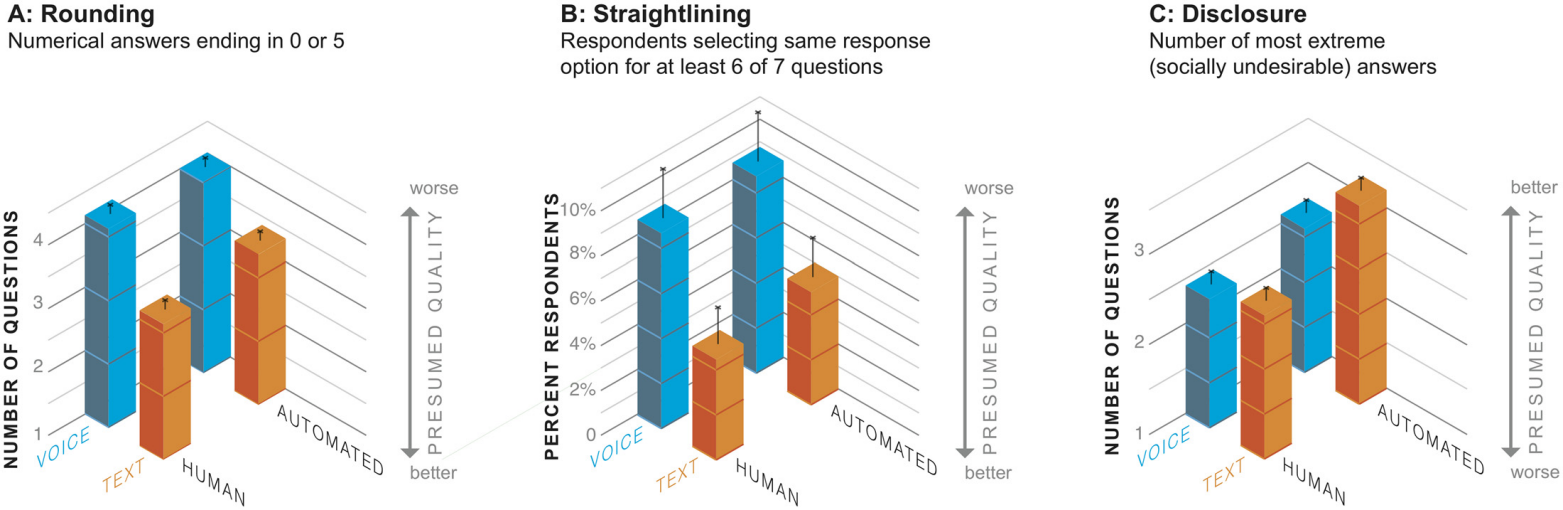
Data quality across the four modes: (A) rounding, (B) straightlining, and (C) disclosure.

People’s answers were more precise in text than in voice interviews (see Fig 3A). Across the 8 relevant questions, respondents provided fewer rounded answers (numerical answers that ended in 0 or 5) in text than in voice interviews, *F*(1,632) = 41.25, *p* < .0001. The amount of rounding was not affected by the interviewing agent (human vs. automated interviewer), *F*(1,632) = 0.55, *ns*, nor did agent interact with the medium of interviewing (text vs. voice), interaction *F*(1,632) = 2.49, *ns*.

**Fig 4:**
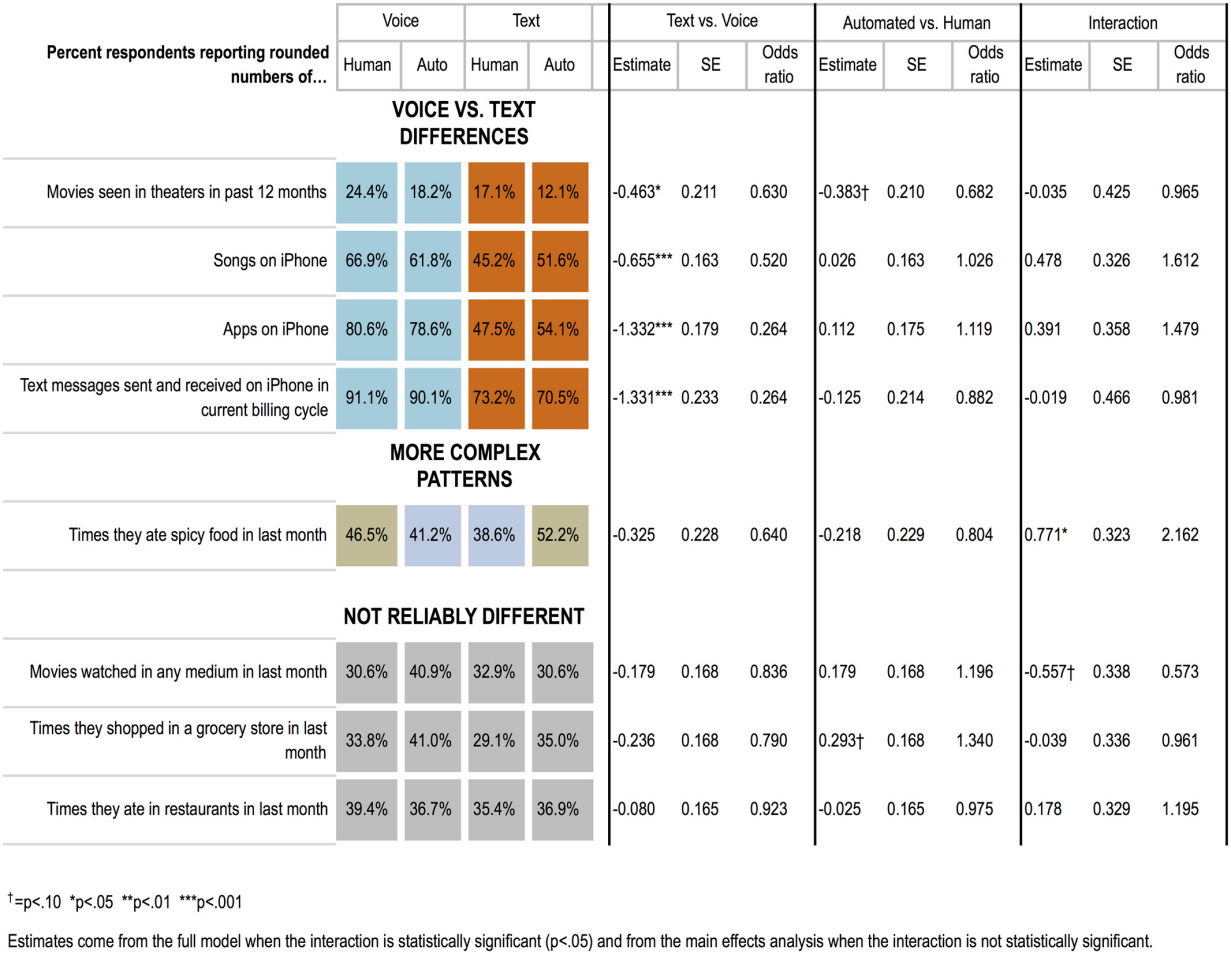
Rounding effects for each question.

For a more fine-grained view of the rounding pattern, see Fig 4; reliably fewer respondents rounded their numerical responses on the questions about movies seen in a movie theater in the past 12 months, number of songs on their iPhone, number of apps on their iPhone, and number of text messages in their most recent billing cycle. For these questions the differences in percentages of respondents rounding in voice vs. text were quite large, from 12 to more than 30 percent; for some questions the more precise answers in text would lead to different substantive conclusions about the population than answers in voice, if the results were used to make population inferences (which of course is not advisable here, given our convenience sample of participants). For example, the average number of apps on respondents' iPhones reported via text was nearly 20 percent higher (50.7 apps) than via voice (42.8 apps), F(1,630) = 4.44, p < .05, and the average number of songs on respondents' iPhones reported via text was 30% lower (479.9 songs) than via voice (692.0 songs), F(1,627) = 3.93, p < .05. We can only speculate about the exact mechanisms underlying these effects, but the findings provide clear evidence that text interviewing promotes more precise responding than voice, independent of whether the interviewing agent is human or automated.

People were also reliably less likely to straightline in text than in voice (see Fig 3B). Across the 7 relevant questions, fewer respondents were likely to select the same response option 6 or more times in text. In a logistic regression, the odds of straightlining in text were about half the odds of straightlining in voice (odds ratio [OR] 0.52, 95% confidence interval [CI], 0.27–0.99, *p* = 0.047). There were no differences by interviewing agent nor did agent interact with medium. These findings again suggest that text interviewing promotes more conscientious responding.

### Data quality: Disclosure

People reported more socially undesirable behaviors in text than voice interviews, and in automated than human interviews (see Fig 3C). This was evident in several ways. Across the 15 relevant questions, respondents produced more answers in text that we determined to be the most socially undesirable, either above the top decile for continuous numerical responses (number of lifetime sex partners, drinks per week, etc.) or the most extreme categorical response option in the stigmatized direction (e.g., exercising less than one day per week, never attending religious services, or having smoked more than 100 cigarettes in one’s entire life), F(1,632) = 7.87, p < .01. People also reported more socially undesirable behaviors to automated than human interviewers, *F*(1,632) = 7.46, *p* < .01. There was no interaction between medium and interviewing agent, *F*(1,632) = 1.45, *ns*.

**Fig 5:**
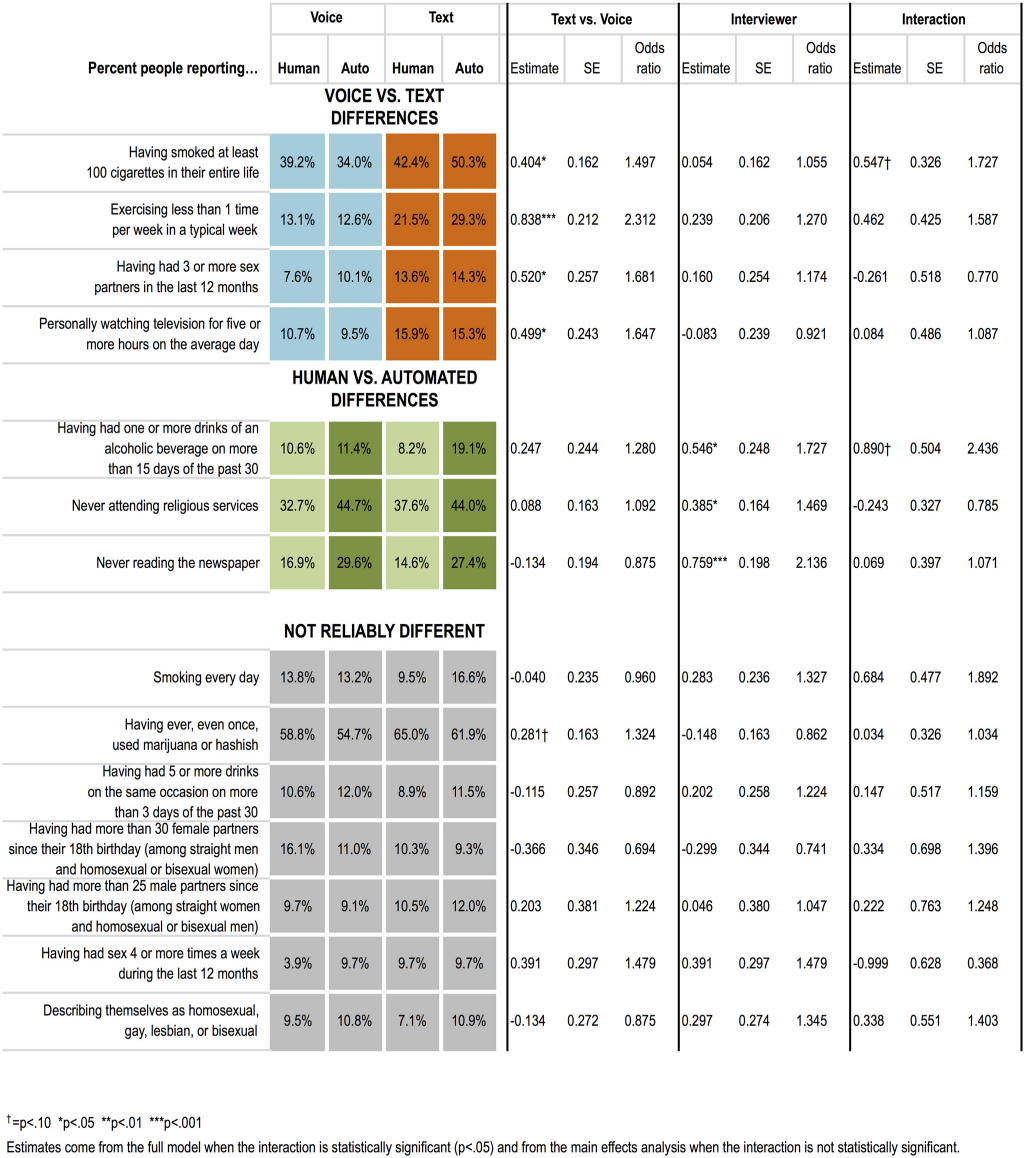
Disclosure effects for each question.

A more fine-grained view of the pattern (Fig 5) shows the percentage of respondents providing the most socially undesirable responses on individual questions. The effects are item-specific, with reliably more disclosure in text than voice for some questions (cigarettes, exercise, number of sex partners, television watching) and more disclosure to the automated interviewer than human for others (drinking frequency, religious attendance, newspaper reading). For these questions the differences in percentages of respondents reporting the most socially undesirable responses are large enough across modes, from 5 to 16%, to affect substantive interpretation and population estimation; for example, if we were to generalize to the population from text interviews in our convenience sample (again, which one would not do in practice), we would estimate that 46.4% of the population has ever been a smoker, compared with 36.6% in voice interviews. Similarly, estimates of how sedentary the population is from text interviews (25.4% exercising less than once a week) would look different than from voice interviews (12.9% exercising less than once a week).

Of course, to understand this phenomenon more fully, one would need to know more about the norms our participants subscribe to, for example, how much exercise they think is desirable in the eyes of others, how embarrassed they are about smoking, how many sex partners they find embarrassing or how important they feel it is to attend religious services. Nonetheless, the fact that our participants were randomly assigned to interviewing modes means that the distribution of norms was unlikely to have differed across the modes, and that the modes were therefore responsible for the levels of disclosure.

These patterns of disclosure replicate—on a mobile device—the well-known effect of greater disclosure to automated systems compared to human interviewers [31], [10], [21]; clearly our participants could tell the difference between human and automated interviews in voice (where the speaker’s being live or recorded is unmistakable) and text (even though the difference is more subtle). They also demonstrate that people disclose more in text than voice, consistent with Hancock et al.’s [32] findings of reduced deception in textual vs. spoken modes of communication. The effects on disclosure of answering textually and answering to an automated interviewer are statistically independent, which suggests that prior findings of greater disclosure in textual automated systems (e.g., web surveys) may have resulted both from automation and also from not speaking.

### Multitasking and mobility

**Fig 6:**
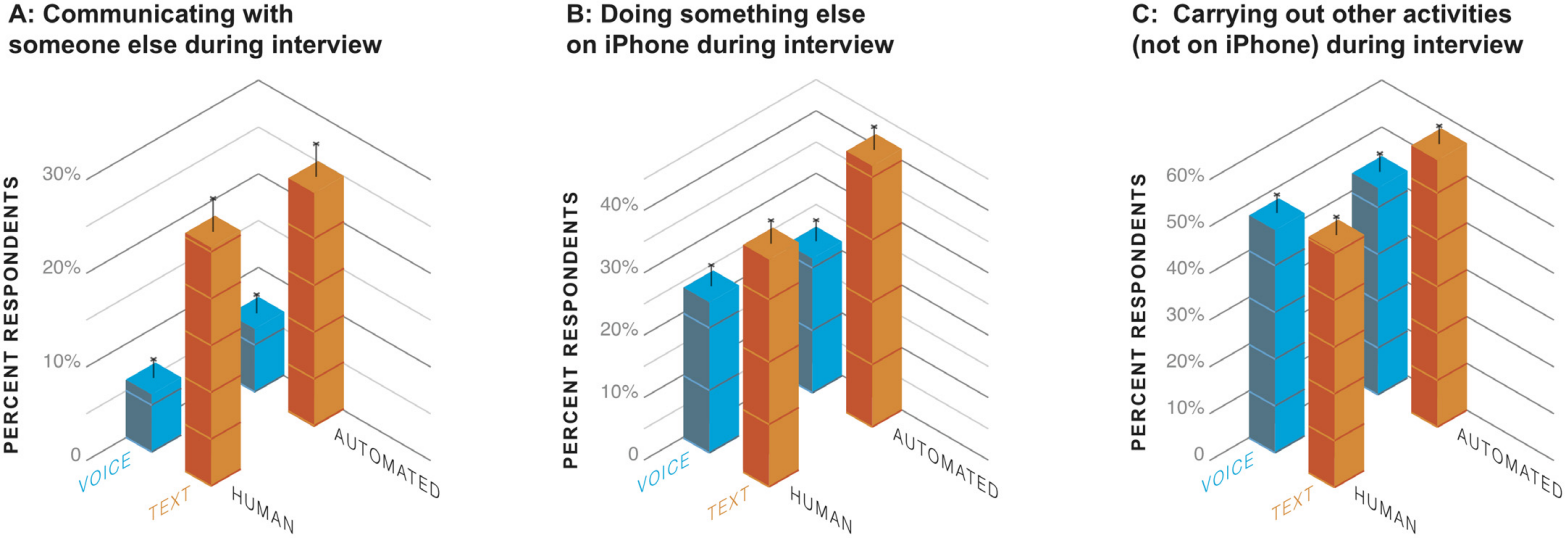
Multitasking across the four modes.

A survey on a mobile multimodal device raises the possibility that respondents might be doing many other things while answering than would have been likely in landline or desktop web surveys. We measured the extent to which this happened in the post-interview online debriefing questionnaire. In general, respondents were more likely to have multitasked during text than voice interviews. As Fig 6A shows, more text respondents reported having communicated with someone else during the interview: either to have talked with someone else on the phone or face to face, to have texted with someone else, or to have videochatted with someone else (OR 4.75, 95% CI 2.85–7.92, *p* < .0001). Respondents in both text modes were more likely to report having carried out any other device-based activities (Fig 6B): watching TV or video, playing video games, or doing anything else on their iPhone or on another device (OR 2.18, 95% CI 1.54–3.08, *p* < .0001). And respondents in both text modes reported having been more likely to be participating in any other activities during the interview (Fig 6C): preparing a meal, eating or drinking, traveling in a vehicle or on foot, working, doing household chores, and shopping or running errands (OR 1.37, 95% CI 1.00–1.88, *p* = .047). There were no effects of the interviewing agent nor any interactions with the medium.

One consequence of being mobile is that there can be variation in respondents’ auditory and visual environments, as well as in whether they are alone or not. Despite being interviewed on mobile devices, most respondents (88%) reported that they were not mobile during the interview. There was also no evidence that respondents in the different modes were any more or less likely to have been in motion (in a vehicle or on foot). Nonetheless, respondents in different modes were differently affected by their circumstances; more respondents in the voice modes reported that background noise had interfered with the interview (29%) than in the text modes (12%) (OR 0.19, 95% CI 0.10–0.36, *p* < .0001), particularly in the automated voice mode (OR 0.36, 95% CI 0.15–0.85, *p* = .020). There were no differences in reported effects of visual glare.

We saw no evidence that reported multitasking by respondents had any impact on the quality of their answers: on their rounding, straightlining, or disclosure. Presumably the asynchronous nature of text messaging allowed multitasking respondents to fully switch their focus between the survey task and other activities, thus allowing them to fully attend to the survey task at the moments they were responding.

### Interview duration and rhythm

**Fig 7:**
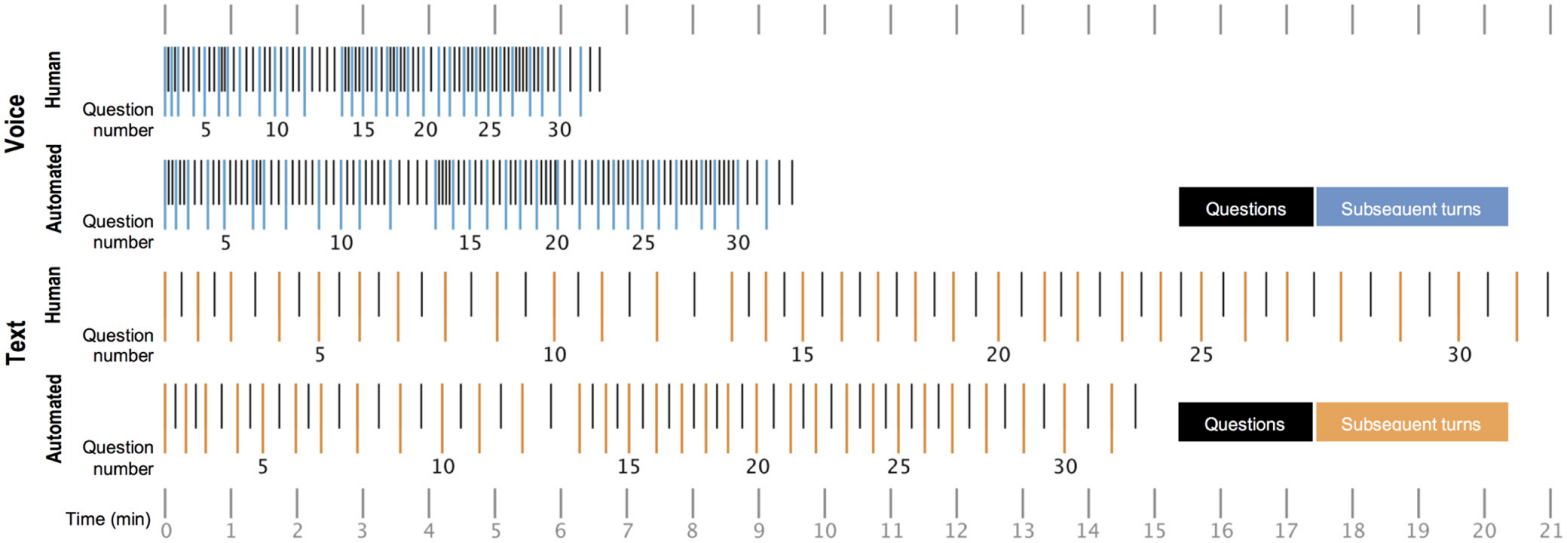
Interview duration and median number of turns per survey question. These timelines display the median duration of question-answer sequences with the median number of turns after each question.

As one might expect, interviews via text had an entirely different rhythm than interviews via voice: they were far longer and less “dense,” with fewer conversational turns after survey questions, than were voice interviews (see Fig 7). Text interviews took substantially longer to complete than voice interviews (Mann-Whitney *U*, *z* = -19.02, *p* < .0001) but they involved reliably fewer conversational turns (*F*[1,617] = 1297.01, *p* < .0001), reflecting a slower back-and-forth compared to the relative rapid fire of voice (as measured by turns per minute—*F*[1,617] = 3111.75, *p* < .0001). (Degrees of freedom for these comparisons reflect that 13 voice interviews were not fully audio-recorded and 2 human text interviews were each missing one sequence). The number of turns following each question in voice was more variable than in text, where most questions were answered in a single turn (Levene’s test: *F*[1,617] = 86.37, *p* < .0001). Questions 7–12, which asked about sexual behavior and orientation, averaged longer response times than other questions in all four modes, presumably due to their sensitivity [33].

Spoken communication clearly differs from communicating by text, with higher attentional demand (strong demand to remain focused) and a requirement to articulate answers (as opposed to entering a single character). Whether the text interviews reflect less total time “on task” is unclear. The fact that text respondents answered more precisely and with more careful consideration suggests that at least some of the time between turns was spent constructing better answers—perhaps counting rather than estimating the frequency of their behaviors, or looking up precisely how many songs or apps they had. A more detailed view of the text interviews suggests that text promotes precision because it imposes less time pressure than voice: respondents gave more precise answers (rounded less) in text interviews that were slower (that is, time between the onset of successive turns was greater than the median of 15.8 seconds) compared with more rapid-fire text interviews (time between onset of successive turns less than the median) (*F*(1,311) = 17.14, *p* < .0001). This effect is specific to text; there was no such difference for slower and faster voice interviews (interaction *F*(1, 628) = 218.73, p < .001), perhaps because the median time between the onset of successive turns in voice interviews was much faster, 4.04 seconds.

These differences in the dynamics of text vs. voice interviews raise the question of which medium is more efficient—either operationally, for interviewers and researchers, or for respondents’ subjective experience of being burdened with an interview. Traditionally, effort and burden have been measured mainly by looking at total elapsed time, as well as measuring respondent satisfaction or irritation [34]–[36]. In mobile text interviews, which allow respondents greater control over how, when and where they respond to each question, it is entirely possible that a longer interview could *reduce* burden, or at least not increase it. Our findings are consistent with this idea: despite the fact that the text interviews took longer, at least as many text respondents reported being very or somewhat satisfied with their interview as voice respondents (95% vs. 93%, *χ* ^2^[1] = 0.65, *ns*). Our text respondents overwhelmingly reported that they would prefer future interviews in text than in voice (84% in human text and 90% in automated text), and a substantial percentage of voice respondents (36% for human voice, and 53% for automated voice) reported that they would prefer text interviews in the future.

However one thinks about efficiency, the bottom line is that even though text interviews took longer than voice interviews, they produced data likely to be of better quality than the data from voice interviews: less rounding, less straightlining, and more disclosure.

### What is the role of non-response?

One interpretation of the findings is that texting promotes better data quality because respondents are under less time pressure and feel less social scrutiny than in voice interviews. Could the findings instead be explained by different patterns of starting and finishing interviews across the four modes? That is, were people who were more conscientious (less likely to round their answers or to straightline) or more willing to disclose sensitive information actually less likely to start or finish interviews in voice modes than in text modes? Could our mode effects result not from the contribution of responders and completers, but instead from the non-contribution of non-responders and non-completers?

Our study design allows us to examine this in a focused way, because all our sample members had already indicated, by screening into the study, interest in and at least some commitment to participating in an interview on their iPhone (in an unspecified interview mode). The fact that our participants were randomly assigned to an interviewing mode means that their initiative was unlikely to have differed across the modes. Thus any mode differences in starting or finishing the interview are unlikely to be due to differences in inclination to participate.

With respect to starting the interview, we see no evidence that different kinds of people (age, gender, ethnicity, race, education, income) in our sample were any more or less likely to participate in one mode than another; see Table 3. Of course, it is possible that variables other than those available to us (age, gender, ethnicity, etc.) differed between those who started voice and text interviews, and thus that those who did not respond to voice (or text) invitations would have answered differently—if they participated—than those who did respond. This would require that the tendency of respondents to give imprecise answers, for example, and the reluctance to engage in a text interview (but willingness to engage in a voice interview) would have the same origin. It is hard to imagine why this would be the case, particularly given the popularity of texting as a mode of communication.

Could differences in *completion* rate account for the mode differences in data quality? The percentage of completion was no different between text and voice interviews (89% vs 92%; *χ* ^2^[1] = 1.16, *ns*), and so differential rates of completion cannot account for the finding that texting promotes better data quality (see Table 2 for more detail). The rate of completion was higher in human than automated interviews (95% vs 86%, *χ* ^2^[1] = 14.84, *p* < .05), as is typical in comparisons of human- to self-administered surveys (see, e.g., [37], chapter 5, for relevant comparisons). Might this account for the greater disclosure in automated than human interviews? This would require a systematic reversal of the pattern of disclosure observed for those who completed and for those who broke off. For non-completion to fully account for the difference in the observed rate of disclosure to automated and human interviewers, the 51 people who broke off with automated interviewers would have needed to disclose 98% less than those who completed the interview (averaging 0.05 instead of 2.91 socially desirable answers). Such a scenario is implausible. If anything, one would expect that those who break off have more to disclose.

## Discussion

People who were randomly assigned to text interviews provided more precise and candid information in survey interviews on smartphones than did people who spoke their answers: they rounded less, they differentiated among response options more (i.e., they straightlined less), and they reported more information that was sensitive. And they did this at rates that would likely affect the substantive conclusions that one would draw from survey data collected in voice versus text interviews, which could in turn affect the policy implications of the data. While of course we can’t be sure that this different pattern of responding reflects greater accuracy, it is reasonable to assume that it does, both on logical grounds and consistent with prior empirical evidence that unrounded answers to objective questions like ours are more accurate [8] and that increased reporting of socially undesirable behaviors is also more accurate [10].

Text interviews also had a fundamentally different dynamic than voice interviews: texters took fewer “turns” spread over a longer period of time, and they were more likely to be doing other things at the same time as the interview. Texting may allow people to be more thoughtful and precise because it is asynchronous, allowing them to take all the time they need and to answer when it is convenient for them—perhaps even giving them time to base their answers on records they have checked (e.g., their log of text messaging this month, or the number of songs on their iPhone). Texting may also make it easier for people to report embarrassing information, since texters do not have to experience the (vocal) presence of an interviewer and (as implemented in our experiment) they didn’t have to speak (or even type) an embarrassing answer. Texters disclosed more even though text data are more persistent and potentially visible to others than voice data.

Independent of the texting effects, people disclosed more sensitive information to automated than to human interviewers. And their precision in responding to automated interviewers was no less than to human interviewers; we saw no evidence that people were less motivated to answer carefully when automated interviewers asked the questions. These findings replicate on mobile multimodal devices the well-known increases in disclosure that occur with self-administered questionnaires [31], [10], [21]. This suggests that the privacy concerns that promote disclosure with automated systems are still relevant in a mobile environment.

These findings raise a number of questions about how these new communication modes can best be used for social measurement. One question is whether the effects of mode of interviewing on conscientious responding and disclosure observed here will replicate across respondents of different ages, income levels, education, technological sophistication, and other individual and group differences. Another is whether the effects change for a sample representing a national population, or even a national population of smartphone users, as opposed to our volunteer convenience sample. Of course, because each mode consisted of a bundle of features, additional research would be needed to determine which particular features or combinations of features are responsible for the effects observed here. Different implementations of each mode, and more choices—for example, the opportunity to answer in a mode other than the contact mode, or even to change interview mode mid-stream—might also lead to different patterns of results.

Nonetheless, the consistency of the patterns and the size of the differences observed lead to clear interpretations, and we believe that the implications will generalize even as technologies develop (our findings represent a snapshot of a particular state of transition in people’s use of evolving communication technologies). First, asynchronous communication—which is here to stay on mobile devices—reduces time pressure to respond and increases social distance with interlocutors, while synchronous communication demands a more immediate response. Whatever new modes may emerge on mobile devices (asynchronous voice communications in enhanced texting applications like WhatsApp are already changing the equation), the implications for social measurement are likely to be the same: more precision and more disclosure are likely to occur in less synchronous modes of interviewing. Whether synchronous (voice, face-to-face) interviews can still be assumed to represent the gold standard for high quality survey data is, as we see it, an open question.

Second, multitasking while answering survey questions does not necessarily lead to poorer data quality, and may well enhance respondents’ satisfaction and well-being by allowing them to respond where and when they find it convenient. In our findings, we do not see evidence of degraded performance from multitasking that is evident in other domains of activity (see [38]–[39]). Even if there are millisecond-level startup and switching costs for respondents when answering survey questions while doing other tasks [40], and perhaps degradation of performance on the other tasks, data quality in our study was actually better in text, where there was more multitasking, than in voice.

Third, automated interviewing on mobile devices increases respondents’ disclosure just as it does in web surveys and other self-administered computerized modes. And the effects of automation are independent of the effects of texting. This suggests that the potential benefits of automation for social measurement extend to the use of a personal portable device despite the varying contexts (public and private) in which the device is used.

Our data do not lead us to argue that all interviews should now be carried out via text messaging or by automated systems. There are likely to be subgroups of the population who would rather not text, and who prefer to speak to a human. Good automated systems have serious development costs (particularly speech systems), which may make them better suited for longitudinal studies where the development costs are amortized, as opposed to one-off or underfunded surveys. But we *are* arguing that, as a larger percentage of the population relies on smartphones for daily communication and computing, people are increasingly likely to expect that they can participate in interviews in more than one mode on their smartphone. As long as people’s self-reports are needed to understand society, researchers should aim to contact and interact with members of the public in ways that best accommodate how they communicate.

## Supporting Information

S1 Fig System diagram. (TIF)

S1 Table Questionnaire items listed in the order they appeared in the survey. (DOCX)
