# Supplementary figures and images for: Precision and Disclosure in Text and Voice Interviews on Smartphones
Source: PLoS One. 2015 Jun 10;10(6):e0128337. doi: 10.1371/journal.pone.0128337 (PMC4465184; doi:10.1371/journal.pone.0128337)

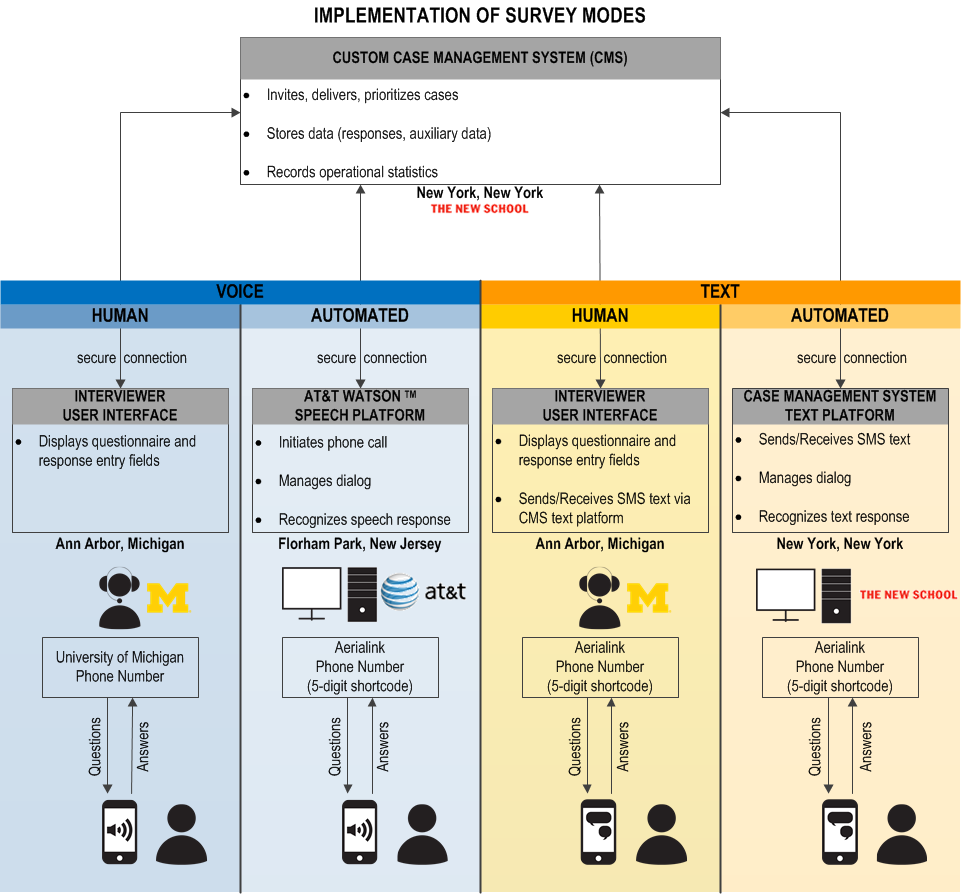

Supplement: S1 Fig — (TIF) [file pone.0128337.s001.tif]
