# Supplementary material for: Precision and Disclosure in Text and Voice Interviews on Smartphones
Source: PLoS One. 2015 Jun 10;10(6):e0128337. doi: 10.1371/journal.pone.0128337 (PMC4465184; doi:10.1371/journal.pone.0128337)
Supplement: S1 Table — (DOCX) [file pone.0128337.s002.docx]

**S1 Table. Questionnaire items listed in the order they appeared in the survey.**

| Order | Question presentation in voice* | Question presentation in SMS text* | Survey of Origin | Sensitive | Numeric | Battery  question |
| --- | --- | --- | --- | --- | --- | --- |
| 1 | How often do you now smoke cigarettes: 'every day', 'some days' or 'not at all'? | How often do you now smoke cigarettes?  A. Every day B. Some days C. Not at all | BRFSS^a1^ | X |  |  |
| 2 | Have you smoked at least 100 cigarettes in your entire life? Yes or no. | Have you smoked at least 100 cigarettes in your entire life? Y or N. | BRFSS | X |  |  |
| 3 | Have you ever, even once, used marijuana or hashish? Say yes or no. | Have you ever, even once, used marijuana or hashish? Y or N. | NSDUH^a2^ | X |  |  |
| 4 | During the past 30 days, on how many days did you drink one or more drinks of an alcoholic beverage? | During the past 30 days, on how many days did you drink one or more drinks of an alcoholic beverage? | NSDUH |  | X |  |
| 5 | During the past 30 days, ...on how many days did you have 5 or more drinks on the same occasion? | During the past 30 days, on how many days did you have 5 or more drinks on the same occasion? | NSDUH |  | X |  |
| 6 | In a typical week, about how often do you exercise? Less than 1 time per week, 1 or 2 times per week, 3 times per week, or 4 or more times per week? | In a typical week, about how often do you exercise?   A. Less than 1 time per week B. 1 or 2 times per week C. 3 times per week D. 4 or more times per week | NHIS^a3^ | X |  |  |
| 7 | How many sex partners have you had in the last 12 months? | How many sex partners have you had in the last 12 months? | GSS^a4^ | X |  |  |
| 8 | During the past 12 months, have your sex partners been 'exclusively male', 'exclusively female', 'both male and female' or have you had 'no partners'? | During the past 12 months, have your sex partners been ...  A. Exclusively male,  B. Exclusively female,  C. Both male and female, or  D. Have you had no partners? | GSS | X |  |  |
| 9 | About how often did you have sex during the last 12 months? 'Not at all', 'Once or twice', 'About once a month', 'two or three times a month', 'about once a week', 'two or three times a week', or 'four or more times a week'. | About how often did you have sex during the last 12 months?   A. Not at all B. Once or twice C. About once a month D. Two or three times a month E. About once a week F. Two or three times a week G. Four or more times a week | GSS | X |  |  |
| 10 | Now thinking about the time since your eighteenth birthday (including the recent past that you've already told us about), how many female partners have you had sex with? | Now thinking about the time since your eighteenth birthday (including the recent past that you've already told us about), how many female partners have you had sex with? | GSS |  | X |  |
| 11 | Thinking about the time since your eighteenth birthday (including the recent past that you've already told us about), how many male partners have you had sex with? | Thinking about the time since your eighteenth birthday (including the recent past that you've already told us about), how many male partners have you had sex with? | GSS |  | X |  |
| 12 | Now I'm going to read a list of terms that people sometimes use to describe themselves: "A" heterosexual or straight; "B" homosexual, gay or lesbian; and "C" bisexual. Which option best describes how you think of yourself? | Here is a list of terms that people sometimes use to describe themselves.  A. Heterosexual or straight  B. Homosexual, gay or lesbian  C. Bisexual  Which option best describes how you think of yourself? | GSS | X |  |  |
| 13 | Now I'd like to ask you some questions specific to your diet. [pause] For the next group of questions, I'll ask you how much you favor or oppose engaging in certain behaviors. After I say the behavior, please say one of the following: strongly favor, somewhat favor, neither favor nor oppose, somewhat oppose or strongly oppose. The first behavior...avoiding fast food. | Now I'd like to ask you some questions specific to your diet. For the next group of questions, I'll ask you how much you favor or oppose engaging in certain behaviors. After I list the behavior, please choose one of the following:   A. Strongly favor B. Somewhat favor C. Neither favor nor oppose D. Somewhat oppose E. Strongly oppose  The first behavior: Avoiding fast food. | Tourangeau et al. 2007^a5^ |  |  | X |
| 14 | The next behavior: Maintaining a healthy diet. | The next behavior: Maintaining a healthy diet. | Tourangeau et al. 2007 |  |  | X |
| 15 | The next behavior... 'monitoring cholesterol levels closely' | The next behavior: Monitoring cholesterol levels closely. | Tourangeau et al. 2007 |  |  | X |
| 16 | The next behavior... 'emphasizing the taste of food rather than its nutritional value'. | The next behavior: Emphasizing the taste of food rather than its nutritional value. | Tourangeau et al. 2007 |  |  | X |
| 17 | The next behavior... 'paying close attention to the nutritional information on food packaging'. | The next behavior: Paying close attention to the nutritional information on food packaging. | Tourangeau et al. 2007 |  |  | X |
| 18 | The next behavior... 'limiting the amount of red meat in your diet'. | The next behavior: Limiting the amount of red meat in your diet. | Tourangeau et al. 2007 |  |  | X |
| 19 | And finally, the last behavior... 'balancing one's diet across the key food groups'. | And finally, the last behavior: Balancing one's diet across the key food groups. | Tourangeau et al. 2007 |  |  | X |
| 20 | During the last month, how many times did you eat spicy food? | During the last month, how many times did you eat spicy food? | Conrad et al. 1998 ^a6^ |  | X |  |
| 21 | How often do you attend religious services? 'At least once a week', 'almost every week', 'about once a month', 'seldom', or 'never'? | How often do you attend religious services?   A. At least once a week B. Almost every week C. About once a month D. Seldom  E. Never | GSS Gallup 1996 follow-up questions on religious service attendance^a7^ | X |  |  |
| 22 | How often do you read the newspaper? 'Every day', 'a few times a week', 'once a week', 'less than once a week', or 'never'? | How often do you read the newspaper?   A. Every day B. A few times a week C. Once a week D. Less than once a week E. Never | GSS | X |  |  |
| 23 | On the average day, about how many hours do you personally watch television? | On the average day, about how many hours do you personally watch television? | GSS |  | X |  |
| 24 | During the last month, how many movies did you watch in any medium? | During the last month, how many movies did you watch in any medium? | New question for this study |  | X |  |
| 25 | During the past 12 months, how many movies have you seen in movie theaters? | During the past 12 months, how many movies have you seen in movie theaters? | New question for this study |  | X |  |
| 26 | During the last month, how many times did you shop in a grocery store? | During the last month, how many times did you shop in a grocery store? | Conrad et al. 1998 |  | X |  |
| 27 | During the last month, how many times did you eat in restaurants? | During the last month, how many times did you eat in restaurants? | New question for this study |  | X |  |
| 28 | How many songs do you currently have on your iPhone? | How many songs do you currently have on your iPhone? | New question for this study |  | X |  |
| 29 | How many apps do you currently have on your iPhone? | How many apps do you currently have on your iPhone? | Pew Internet & American Life Project (sep.2010)^a8^ |  | X |  |
| 30 | How many text messages have you sent and received on your iPhone in your current billing cycle? | How many text messages have you sent and received on your iPhone in your current billing cycle? | New question for this study |  | X |  |
| 31 | Finally, we have two last questions on earlier topics in the interview. Let's return to smoking. This time be sure to include any puffs on any cigarettes, whether or not you inhaled AND whether or not you finished them. Keeping this in mind, have you smoked at least 100 cigarettes in your entire life? Yes or No. | Finally, we have two last questions on earlier topics in the interview. Let's return to smoking. This time be sure to include any puffs on any cigarettes, whether or not you inhaled AND whether or not you finished them. Keeping this in mind, have you smoked at least 100 cigarettes in your entire life? Y or N. | BRFSS |  |  |  |
| 32 | And for the last question, let's return to newspapers. This time be sure to consider any newspaper content no matter whether it appears in print, online or on a mobile device. And be sure to count only newspapers from recognized journalistic outlets. Keeping this in mind, how often do you read the newspaper? 'Every day', 'a few times a week', 'once a week', 'less than once a week', or 'never'? | And for the last question, let's return to newspapers. This time be sure to consider any newspaper content no matter whether it appears in print, online or on a mobile device. And be sure to count only newspapers from recognized journalistic outlets.   Keeping this in mind, how often do you read the newspaper?   A. Every day B. A few times a week C. Once a week D. Less than once a week E. Never | GSS |  |  |  |
| * In both voice modes, respondents were required to articulate the full response option wording, except for Q12 where they could answer with just the letter (A, B, or C) because the full response option wording was so potentially revealing. In the auto text mode, respondents were required to type the option letter (i.e. A, B, C, D); in the human text mode, people could either type the option letter or the full response wording if they chose.  Note: Two questions (Q1 and Q22) were re-asked but with definitions for terms that might have been ambiguous (Q31 and Q32), to allow testing whether interview mode had affected interpretation of or clarification seeking in the previous questions. | | | | | | |
| ^a1^ BRFSS: Behavioral Risk Factor Surveillance System survey ^a2^ NSDUH: National Survey on Drug Use and Health ^a3^ NHIS: National Health Interview Survey ^a4^ GSS: General Social Survey ^a5^ Tourangeau, R, Couper, MP, Conrad, F (2007) Color, labels, and interpretive heuristics for response scales. *Public Opinion Quarterly*, *71*(1), 91-112. ^a6^ Conrad, G, Brown, NR, Cashman, ER (1998) Strategies for estimating behavioural frequency in survey interviews. *Memory, 6*(4), 339-366.  ^a7^ In U.S., Four in 10 Report Attending Church in Last Week. (2013, Dec.24). *Gallup Trends A-Z.* Retrieved from http://www.gallup.com/poll/166613/four-report-attending-church-last-week.aspx  ^a8^ Purcell, K, Entner, R, & Henderson, N (2010, Sept. 14) The Rise of Apps Culture. Pew Internet & American Life Project. Retrieved from <http://www.pewinternet.org/2010/09/14/the-rise-of-apps-culture/> | | | | | | |
|  | | | | | | |
